# Developing occupational therapy students’ information and historical literacy competencies: an interprofessional collaborative project

**DOI:** 10.5195/jmla.2018.332

**Published:** 2018-07-01

**Authors:** Rita P. Fleming-Castaldy

**Affiliations:** Professor, Department of Occupational Therapy, University of Scranton, Scranton, PA

## Abstract

**Objective:**

The study examined the efficacy of an interprofessional information and historical literacy project implemented by an occupational therapy educator and a librarian.

**Methods:**

A graduate course was revised to include information and historical literacy objectives and instruction. A course-specific questionnaire administered on the first and last day of class, assignment grades, and course evaluations provided measures of project outcomes for six years. Differences between questionnaire pre- and post-test means were determined using *t*-tests. Course evaluation comments were analyzed to obtain qualitative perceptions.

**Results:**

A significant difference (*p*<0.0001) was found between pre-test (M=3.93, SD=0.48) and post-test (M=4.67, SD=0.30) scores of total information and historical literacy competence across all years (n=242). Responses to individual items also differed significantly (*p*<0.0001). Student ratings (n=189) from the course evaluation historical literacy objectives were high (M=4.6 on a 5-point scale). Assignment quality and grades improved, and course evaluation comments reflected student satisfaction.

**Conclusions:**

The findings supported the hypothesis that students’ self-reported information and historical literacy competencies would increase after project participation. Acquired skills were evident in students’ assignments. Research to determine if these capabilities were used post-graduation is needed. Because this was a course-specific project, findings are not generalizable; however, the instructional methods developed for this project can serve as a model for effective interprofessional collaboration. The broadening of information literacy instruction to include discipline-specific historical literacy provides a unique opportunity for health sciences librarians and educators. Developing students’ historical literacy in their chosen fields can help them understand their profession’s present status and be informed participants in shaping its future.

## INTRODUCTION

Founded in 1917, occupational therapy is a dynamic profession with a 100-year history of meeting societal needs [1, 2]. From its inception to the present, its practitioners have used therapeutic occupations to promote health and wellness and enable people with physical, cognitive, and/or psychiatric conditions to fully participate in society [3, 4]. Over the course of its first century, the field has been influenced by historical events (e.g., World Wars, the Disability Rights Movement) and paradigm shifts (e.g., medical paternalistic models versus person-directed empowerment models) [5–8].

For decades, occupational therapy’s leaders have advocated for practitioners to be cognizant of how the profession’s history determined its evolution. [9–13]. For example, Gillen described how choices made during the profession’s development led away from its philosophical base, which is grounded in the therapeutic use of occupation [14]. He analyzed decades of evidence that supported a return to occupational therapy’s roots and urged practitioners to choose approaches that were consistent with its founding principles rather than reductionist methods. Colman [15] and Freidland [16] also examined this shift and the power differentials that occurred during the 1940s and 1950s, when physicians sought to control occupational therapy education and service provision. The lessons learned from the past are evident today in occupational therapy’s promotion of its autonomy and unique value in a rapidly changing practice environment [2, 17].

The major events and practice shifts in occupational therapy’s historical timeline, such as those noted above, are well documented in the profession’s core textbooks [1, 18, 19]. However, simply knowing recorded events is insufficient. Content knowledge does not ensure literacy [20]. To be historically literate, a person must have “the ability to glean appropriate information about the past from resources of many genres (and) the ability to engage in historical processes—to not simply possess knowledge, but to know how to build it” [21]. This definition is congruent with long-standing calls in occupational therapy’s literature to use the field’s history to understand the present and inform its future [9–16]. In their 1977 seminal analysis of the profession’s first sixty years, Kielhofner and Burke concluded, “The history of occupational therapy, the interpretations of events and contexts leading up to the present provides a perspective from which to make some predictions about what is needed to ensure the future of occupational therapy” [9]. More recently, Molke challenged the profession to critically examine the dominant philosophies that structured how its history has been routinely conceptualized [22].

Several scholars accepted Molke’s challenge, and occupational therapy’s history is now more inclusive of social, political, and cultural contexts that impacted its development [23–26]. However, these works are narrative and philosophical. There are no published guidelines on *how* to help occupational therapy students become historically literate [27]. To attain occupational therapy’s historical literacy aspirations, this pedagogical gap must be addressed. This need is especially vital for students who were born after the major historical events that impacted the profession. Current American post-secondary students’ primary and secondary education were based on legislated standards (e.g., No Child Left Behind Act) that emphasized reading and mathematics and marginalized social studies [28, 29]. Subsequently, many are ill informed about history [29, 30]. Given that today’s students will chart occupational therapy’s future path, I believe educators have a responsibility to address this lack of knowledge.

In this paper, I describe a six-year study that examined the efficacy of a course-specific interprofessional project designed to develop occupational therapy students’ historical literacy competencies. Because information literacy (IL) is the foundation of historical literacy [30–32] and competence in IL is essential for meeting current and future practice demands [33–38], the development of core IL competencies was embedded in this project. Project objectives included developing students’ abilities to identify a scholarly journal, access needed information effectively and efficiently, and understand the difference between primary, secondary, and tertiary sources.

This project also sought to develop students’ abilities to access and use information ethically and legally, identify current and historical literature and resources that are appropriate to meet the course requirements, and effectively manage and use acquired information and resources to complete course assignments. Additional historical literacy objectives included developing students’ abilities to recognize the difference between historical literature and clinical research and articulate the value of acquiring a historical perspective of occupational therapy to their professional development.

The hypothesis for this project was that students’ self-report of competence in information and historical literacy will be higher after they participate in course-specific information and historical literacy teaching-learning activities.

## METHODS

### Background

Interprofessional collaborative teaching-learning models that embed IL into curricula in an active, integrated manner are more effective than passive, fragmented, single-session class presentations [38–45]. Recognizing the well-documented value of infusing IL into curricula and the established efficacy of interprofessional faculty collaboration [38–45], the University of Scranton Weinberg Memorial Library implemented an initiative to encourage and financially support this process in 2005. This library-funded program awards an average of 5 $1,000 stipends per year to faculty members who partner with an assigned discipline-specific librarian to develop and execute a course-specific IL project.

In 2010, I joined this IL initiative and received a stipend for a fall semester first-year graduate course, “Leadership in Occupational Therapy.” This course’s capstone project requires students to identify a personal interest in a specific aspect of occupational therapy. They then complete historical research about their self-selected topics from the inception of occupational therapy to the present. Based upon an extensive literature review, students compose a reflective analysis of the profession’s evolution and the impact of external and internal contexts on this trajectory. Supplemental Appendix A outlines the assignment’s main criteria and grading rubric. The successful completion of this critical historical research analysis requires information and historical literacy.

While students’ undergraduate education included IL instruction, their required assignments typically had clear structure and faculty-assigned foci (e.g., answering a person, intervention, comparison, outcome [PICO] question). The independent research expectations and self-directed nature of the graduate capstone project presented students with a new experience. In response, many students verbally expressed self-doubt about their abilities to meet course standards. Librarians and I provided individualized guidance, but this one-on-one approach depended on students’ initiative to seek assistance. A more formal and systematic approach to develop students’ knowledge and skills was needed. The acquisition of a library IL stipend enabled us to address this need.

Working with the occupational therapy discipline–specific librarian, I revised the existing course to increase its focus on developing students’ information and historical literacy competencies. Supplemental Appendix B summarizes project-specific course objectives and instructional methods. To implement these educational approaches, the librarian and I co-taught two class sessions per semester. Each two-hour-and-forty-minute session included a brief formal instruction followed by experiential activities and guided hands-on work to attain specific project objectives. The first session was conducted during the first or second week of class and the second during the fifth or sixth week of class. In between these “lab” sessions, the weekly class seminars included time to discuss students’ progress with their assignments and analyze their findings in the historical contexts of different time periods. Individual one-on-one meetings provided additional guidance throughout the semester.

To decrease the complexity of students’ historical research and ease their completion of their capstone project, the librarian developed a course-specific web-based research guide (supplemental Appendix C).

### Data collection

The time frame for this project’s data collection was 2010 to 2016. In the summer of 2010, the discipline-specific librarian and I developed a ten-item questionnaire based on the IL standards of the Accreditation Council for Occupational Therapy Education [46] and the American College & Research Libraries [47], and course-specific requirements. Because this course emphasized how occupational therapy’s leadership history influences the profession’s present and future, three items focused on historical literacy competencies.

This questionnaire was not inclusive of all IL competencies. We purposefully decided to limit items to increase response rate [44] and to focus on the core competencies needed to attain project goals. The face validity of this questionnaire was established by basing it on published IL standards, course criteria, and peer review by occupational therapy and library faculty. Because the stipend supporting this project required its implementation the semester after it was granted, the discipline-specific librarian and I did not complete formal reliability or validity studies.

The paper questionnaire was administered as a project pre-test on the first day of class and administered again as a project post-test on the last day each fall semester from 2010–2016. Students were asked to rate each item according to a 5-point Likert scale (1=definitely disagree, 2=somewhat disagree, 3=unsure, 4=somewhat agree, 5=definitely agree). No identifying information was requested, and students were given no incentives to participate. Additional quantitative data were obtained by reviewing students’ submitted assignments according to objective grading criteria and student-completed university course evaluations.

At the University of Scranton, instructors can add course-specific objectives to the university’s online course evaluations. The historical literacy objectives added to the course evaluation for this project were “I can articulate the value of an historical perspective to my profession of occupational therapy”and “I know how to identify and access historical information and resources appropriate to meet the course requirements.” Students’ agreement with university course objectives were rated according to a 5-point Likert scale (1=strongly disagree, 2=disagree, 3=neutral, 4=agree, 5=strongly agree). Students could also select “NR” to indicate no response to an item. Qualitative data about student perceptions of the course and its teaching-learning activities were collected from the comments section of the course evaluations.

### Data analysis

Course evaluation and questionnaire data were analyzed as an aggregate. Descriptive statistics determined the frequencies and means for the course evaluation items and the pre- and post-test responses to the 10 questionnaire items. A total information and historical literacy competency mean score was obtained by averaging the pre- and post-test means. Differences between pre- and post-test means were determined using *t*-tests, and the effect sizes of these differences were calculated. In addition to the quantitative analysis of student course evaluation ratings, a qualitative analysis of course evaluation comments was completed. All student comments were collated. Each comment in the resulting list of 489 comments was categorized according to its primary focus. Comments that directly related to the information and historical literacy project were analyzed further to identify commonalities and recurring themes.

## RESULTS

Two hundred and fifty-four students completed the course over a period of 6 years; all successfully met the established grading criteria for their capstone projects. Most students completed the course questionnaire (95%, n=242) and the university course evaluation (74%, n=189). Because the questionnaires and course evaluations were done anonymously and students had the right to refuse to participate, their demographics were not collected. However, participants were part of a homogenous group. During this time period, occupational therapy graduate students were 96.5% female (n=245; 3.5% male, n=9) and 97.2% Caucasian (n=247; 2.8% non-Caucasian, n=7). Only 2.4% (n=6) were older than 25 years.

Course evaluation ratings for attaining course-specific historical literacy objectives were high (mean [M] =4.6 on a 5-point scale). A significant difference was found between the pre-test (M=3.93, standard deviation [SD]=0.48) and post-test (M=4.67, SD=0.30) total information and historical literacy competence mean scores across all years (*t*(241)=22.79, *p*<0.0001). Student responses to the 10 individual items in the questionnaire also differed significantly between the pre-test and post-test (*p*<0.001). Table 1 provides the mean data for all questionnaire items and their corresponding statistics in the order of largest to smallest effect size between the pre-test and post-test.

**Table 1:** Information and historical literacy competence questionnaire: pre- and post-test results

Information and historical literacy competency	Pre-test	Post-test	Statistics
I know how to identify historical information and resources appropriate to meet the course requirements.	M=3.25 SD=0.85	M=4.78 SD=0.42	*t*(241)=27.18 *p*<0.0001 *d*=2.39
I can articulate the value of acquiring a historical perspective of occupational therapy to my professional development.	M=3.87 SD=0.81	M=4.83 SD=0.41	*t*(241)=17.43 *p*<0.0001 *d*=1.57
I recognize the difference between historical literature and scholarly research.	M=3.31* SD=0.94	M=4.41* SD=0.66	*t*(240)=15.78 *p*<0.0001 *d*=1.38
I know how to identify current information and resources appropriate to meet the course requirements.	M=4.26 SD=0.55	M=4.84 SD=0.39	*t*(241)=13.49 *p*<0.0001 *d*=1.23
I can effectively use information and resources to complete a specific course-related assignment.	M=4.29 SD=0.65	M=4.83 SD=0.38	*t*(241)=11.71 *p*<0.0001 *d*=1.04
I understand the difference between primary, secondary, and tertiary sources.	M=3.49 SD=0.93	M=4.28 SD=0.60	*t*(241)=11.09 *p*<0.0001 *d*=1.03
I can effectively manage the information and the sources gathered to complete course assignments.	M=4.15 SD=0.76	M=4.70 SD=0.48	*t*(241)=9.73 *p*<0.0001 *d*=0.89
I can access needed information effectively and efficiently.	M=4.04 SD=0.65	M=4.52 SD=0.60	*t*(241)=9.31 *p*<0.0001 *d*=0.76
I know how to access and use information ethically and legally.	M=4.38 SD=0.67	M=4.75 SD=0.47	*t*(241)=7.02 *p*<0.0001 *d*=0.65
I know how to identify a scholarly journal.	M=4.26* SD=0.77	M=4.67 SD=0.53	*t*(240)=7.24 *p*<0.0001 *d*=0.63

* n=241; n=242 for all other items.

M=mean.

SD=standard deviation.

Of the 489 comments provided on the course evaluations, 230 (47%) were related to the information and historical literacy project. The other comments related to the instructor (e.g., teaching style, grading criteria, availability) and course logistics (e.g., assignment due dates, class schedule). The analysis of student comments about the information and historical literacy project revealed 5 major themes: the value of teaching-learning activities and course resources, acquired information literacy competencies, acquired historical literacy competencies, enhanced professional identity, and personal pride. Table 2 provides representative student statements for each theme.

**Table 2:** Qualitative analysis of student course evaluation comments

Theme	Representative statements
Value of course assignments	The reflections and discussions made me think about things differently. The historical analysis is a “must have” assignment. I truly felt that I was performing graduate level work.
Acquired information literacy competencies	I learned how to best define search terms and when to use “and” versus “or” when utilizing our library’s numerous databases to maximize search results. I became familiar with helpful databases such as CINAHL, EBSCO, OT Search, and Wiley Online Library that enriched my research collection. I learned how to search through reserves and journals for important information. We learned to use alternative ways to find primary, secondary, and tertiary resources when we felt like our electronic resources had been exhausted.
Acquired historical literacy competencies	We learned a lot about the history of OT and the struggles we have faced to get to where we are today; learned how OT has transformed through the years; learned the issues the profession has faced and how they tackled these obstacles. It’s important for OTs to know and understand some of the issues that have faced our profession so we can be aware and avoid the same mistakes in the future.
Enhanced professional identity	I feel that I really developed a sense of personal responsibility within the profession of OT. I feel like I am more empowered to go into the work force with the ability to make necessary changes. The topics covered were very valuable to my professional development. It was very helpful to discuss the issues that we may come across and how to act as good leaders when challenged by these problems. I feel I am more confident in what my profession is and how to advocate for it; passionate about being a competent therapist.
Personal pride	It was challenging but I was motivated by it’s *[sic]* difficulty because I knew I could accomplish the task and I was motivated to do it well! I am so impressed with what I was actually able to accomplish and impressed with my comprehensive knowledge on my topic. I was very happy with and proud of my work on our primary project.

OT=occupational therapy.

Informal observations of the students in class and during individual meetings provided additional insights into the effectiveness of this information and historical literacy project. During the initial class sessions and meetings, most students expressed apprehension about the capstone course requirement. They exhibited difficulty accessing online resources and asked many questions about how to conduct an effective literature search. As the course progressed, students were observed asking informed questions and engaging in critical discourse about the literature that they acquired and its implications. For example, many were appalled by the terminology that was used in the literature to describe persons with disabilities and the dominance of institutionalization. Students questioned the social basis for terms such as crippled [48–50], imbecile [51], mental cases [52–53], and mental defectives [54] and the pervasive segregation of people with disabilities [55–57]. Many discussions focused on how the disability rights movement changed the perceptions of persons with disabilities and the resulting legislation that enabled their social participation [7, 8, 23–27]. The most frequently asked question was: “Why didn’t we learn disability history in high school?”

Over the course of the semester, students were observed to be more proficient in their searches. They often shared “good finds” with their peers by calling out a source during class labs and/or sharing it via email (e.g., the description of home-based occupational therapy in 1922 [58] and a call for evidence-based practice [EBP] in 1934 [59]). They used diverse resources (e.g., microfilm, interlibrary loan) and expressed confidence and pride in their historical research. This observed competence and reported self-efficacy is supported by student comments on their course evaluations and their attainment of good grades on their capstone projects. According to the assignment’s objective grading criteria, all earned a B or better; most earned As.

## DISCUSSION

The significant differences found between the pre-test and post-test information and historical literacy competence mean scores supported the hypothesis that students’ self-report of competence in information and historical literacy would be higher after their participation in course-specific information and historical literacy teaching-learning activities. The generally large effect sizes found for each questionnaire item supported the magnitude of these differences [60]. According to Cohen, a large effect size is 0.80 and a medium effect size is 0.50 [60]; 7 of the questionnaire items exceeded the first standard and 3 the latter.

The finding that students reported increased IL skills after their participation in this course project was consistent with studies completed with medical [61], physician assistant [62], nursing [42–44, 63], and rehabilitation therapy students [33, 34]. While enhanced confidence does not equate to application of skills [64], there seemed to be a connection in this case. After the implementation of this information and historical literacy project, the majority of students earned As on their capstone projects, and no grade was lower than a B. This was not the case prior to the project’s implementation, when students’ grades on the capstone project varied. Those who consistently sought individualized guidance did well. Students who did not actively seek external help to acquire IL skills struggled with the assignment, and their lower grades reflected this gap.

The students who participated in this IL project completed their coursework in a manner that exhibited solid information and historical literacy skills. Consistent with Nokes’s definition of historical literacy, students integrated the knowledge that they acquired from multiple resources to write coherent and well-substantiated analyses of the evolution of their selected professional domains from occupational therapy’s inception to the present. Students’ submitted papers supported that they had learned “not [to] simply possess knowledge, but to know how to build it” [21]. Their analyses consistently reflected the acquisition of an enriched perspective about occupational therapy and the value of learning (and remembering) lessons from the past. This appreciation greatly enhanced seminar discussions about current and emerging opportunities and challenges to occupational therapy and how students could personally shape the profession’s future. Because perspectives gained from facilitated dialogue can influence students’ future work, this outcome is meaningful. How occupational therapy practitioners interpret problems, approach practice, and forge their professional identities is “shaped partly by the discourses in which they participate” [65].

The success of this IL project can be attributed to the fact that it was analogous to documented best practice. Effective IL education relies on interprofessional collaboration that is based on knowledge of and mutual respect for each other’s fields [33, 39, 41, 66, 67]. From the initial IL stipend proposal, the occupational therapy discipline–specific librarian and I worked together to use our respective professional expertise to design and implement this project. We co-taught IL labs and collaborated to strengthen course resources and teaching-learning activities. We learned from each other and knew when to refer students to the other for specialized knowledge [41]. The discipline-specific librarian also educated other library faculty and staff about this project’s historical literacy objectives and resources. Consequently, they were able to provide informed guidance to students.

When IL education is timed right and directly related to assignments it “makes sense for students” [41]. This project was initiated in students’ graduate year, and the assignments drew on their undergraduate foundational knowledge. Consistent with Lusk’s advice to select topics for historical analysis that are “intriguing and capable of sustaining a researcher’s interest” [68], students were guided in selecting topics that personally interested them. Allowing students to self-determine their courses of inquiry piqued and maintained their interest. Throughout the semester, many openly expressed delight at discovering a key work that related to their topics. Students were observed to be highly engaged when scrolling through microfilm and paging through journals from the pre-digital age.

Discovering that today’s EBP is rooted in the profession’s founding principles (e.g., the use of play activities with children) and finding the first published record of a practice innovation (e.g., dynamic splinting) were particularly exciting. These observations are meaningful for completing historical research, and “The pleasure of the pursuit should not be underrated” [68]. As one student noted in her comments about the efficacy of the course online research guide:

The *Archives of OT* and *Occupational Therapy and Rehabilitation* turned up primary sources which were so interesting and so otherwise unattainable to me. There is also the *Modern Hospital* eJournal which allowed a reader to turn the pages of magazines from the 1910s. Seeing the pages flip just as if you were holding the journal in your hands was an excellent way to hold a reader’s interest and make them keep going, keep digging through the past to try to understand what life was like.

As reflected in the above statement, the rich and robust online course-specific research guide developed by the discipline-specific librarian was an invaluable asset. Being able to easily access relevant resources that reflect the scope of occupational therapy is an identified IL need [69]. To support student research, Cobus-Kou and Waller advocated for developing web-based course guides [67]. The utility of an electronic resource was highly evident in this project. The course-specific research guide supplemented in-class instruction by providing helpful resources for students to complete their research and compose their papers. Using this research guide, students accessed historical archives, seminal works, and current literature encompassing the depth and breadth of occupational therapy with reported ease.

Institutional support for a designated discipline-specific librarian and the acquisition of outstanding library resources is undeniably the most important factor in this project’s success. The openness of library department members in assisting the discipline-specific librarian in providing targeted and informed guidance to occupational therapy students was a definite strength. When institutional and departmental commitment is lacking, the ability to effectively integrate IL education into curricula is hindered [66, 69].

The opportunity to work with and learn from librarians who were astute about IL *and* occupational therapy was recognized by students who frequently commented on the value of personal meetings with librarians. In reflecting on the initial angst that the capstone project induced, one student concluded that her anxiety was unfounded, stating “never fear, the Weinberg Library is here!” Another student noted:

Several librarians were able to assist me during this month-long scavenger hunt for research pertaining to my topic…Their mutual curiosity for your topic and assumption of your research task as if it was their own truly can make even the most unlikely student enjoy the research process and see it as [a] challenging, yet feasible undertaking.

Students desire this helpful and informed approach [69, 70]. Morgan-David and Preston found that when library staff had limited knowledge of occupational therapy, students felt that their information needs were not understood and that provided services were irrelevant [69].

While this information and historical literacy project had positive outcomes, its limitations must be recognized. Because this was a course-specific project implemented with a homogeneous class, the findings are not generalizable. This project’s questionnaire was limited by fact that it was a self-report and not an objective measure of skills [64]. This limitation was balanced by using objective grading criteria to review assignments. All submitted work effectively met these objective outcome standards and demonstrated students’ acquisition of desired competencies. Future research is indicated to determine if these capabilities were sustained and used during students’ subsequent assumption of professional roles as occupational therapists.

While a majority (74%) of students completed the course evaluation, the views of students who did not participate in the university course evaluation process are unknown and thus not represented in this study. Because I was the only person to review the course evaluation comments, the qualitative analysis of student comments is limited to one person’s interpretation. By its nature, qualitative analysis is open to interpretation; therefore, a different researcher reviewing the course evaluation comments may have derived different or additional themes [71]. In retrospect, approval for an external reviewer to participate in the analysis of student comments should have been sought.

Despite these limitations, this interprofessional project was effective. Student responses to a pre- and post-test questionnaire, course evaluation ratings and comments, and informal observations support students’ enhanced self-efficacy in information and historical literacy. Their successful completion of course work objectively demonstrates their acquired competencies.

To further understand students’ acquired historical literacy, an institutional review board–approved content analysis study of their historical capstone projects is underway. This study is being completed with the assistance of 2 external reviewers who are knowledgeable about content analysis procedures. Themes will be identified to determine the lessons that students learned from examining occupational therapy’s 100-year history. Student responses to a survey about their professional and personal “‘take-away” messages from this project will also be analyzed. Ongoing review of students’ coursework and evaluations will continue to inform project development.

The extraordinary technological advances of the last decade and the resulting information explosion require an increased prioritization of the development of students’ IL competencies [31, 35, 61, 62, 72]. In the health sciences, IL provides the foundation for informed clinical reasoning, EBP, professional development, clinical research, and lifelong learning [35–38, 61, 73, 74]. By harnessing the endless resources afforded by technology, health sciences educators have an unparalleled opportunity to partner with librarians to develop resource-rich discipline-specific IL programs. These should include primary resources and archival works, integrated and meaningful learning activities, and assignments that capture self-identified interests. This paper described how an interprofessional collaboration applied these best IL educational practices to purposefully design and implement a project focused on developing occupational therapy students’ historical literacy.

Given that occupational therapy is beginning its next century in a health care environment fraught with uncertainties, the need for historically literate practitioners is great. Collaborating with librarians, occupational therapy educators can use IL instruction to help students learn about the profession’s history. They can facilitate students’ critical analysis of the influences and contexts that formed the field. The resulting historical literacy can strengthen students’ professional identities. The importance of historical literacy to occupational therapy and related professions is underscored by Greenberg who observes, “There are few fields whose development is so deeply tied to its past than the health sciences. History matters to us: we measure our progress from where we started as well as to where we want to go” [75]. The potential for librarians and health care educators to form collaborative partnerships to help students develop and learn to apply historical literacy is an educational opportunity worth seizing. The resulting historical acumen can enable students to become informed participants in forging their respective profession’s future.

## SUPPLEMENTAL FILES

Appendix A Historical literacy capstone project criteria and grading rubric

Appendix B Information and historical literacy learning objectives and instructional methods

Appendix C Contents of the leadership in occupational therapy online research guide
