# Supplementary material for: Developing occupational therapy students’ information and historical literacy competencies: an interprofessional collaborative project
Source: J Med Libr Assoc. 2018 Jul 1;106(3):340–51. doi: 10.5195/jmla.2018.332 (PMC6013127; doi:10.5195/jmla.2018.332)
Supplement: Appendix A [file jmla-106-340-s001.pdf]

JMLA Journal of the Medical Library Association

SC

Supplemental content to
J Med Libr Assoc. Jul;106(3):dx.doi.org/10.5195/jmla.2018.332
www.jmla.mlanet.org
© Fleming-Castaldy 2018

Developing occupational therapy students' information and historical literacy competencies: an interprofessional collaborative project

Rita P. Fleming-Castaldy

APPENDIX A

Historical literacy capstone project criteria and grading rubric

Specific assignment criteria with examples	✓	+	–	0
Articulation of self-selected focus. This can include a leadership domain (e.g., research), a practice area (e.g., mental health), or a population (e.g., veterans).				
Effective use of past terminology to trace the origins of chosen focus (e.g., childhood schizophrenia for autism) and obtain needed sources.				
Synthesis of at least five primary sources to present an organized and integrative description of the history and evolution of selected focus in:				
The founding years: 1910s–1920s				
The 1930s–1940s				
The 1950s–1960s				
The 1970s–1980s				
The 1990s–the present				
Identification of a "thread" in the literature that links the past to the present.				
Coherent, cogent, and well substantiated analysis of:				
• how this "thread" (e.g., legislation, practice models) impacted the historical course of the paper's primary area of focus.				
• the major historical influences (e.g., wars, the Civil Rights movement, technological advances) on selected "thread" and area of focus.				
• how the values, ethics, and/or leadership styles of the past influenced the evolution of selected "thread" and area of focus.				
• how the identified historical influences impact the present status of the selected area of focus.				
• how present values, ethics, and/or leadership styles impact the present status of the selected area of focus.				
• the potential for future growth of the selected area of focus with a substantiated rationale for this conclusion.				
References comply with American Psychological Association standards.				

✓="On target," clear and well-written, fulfills assignment requirement.

+=All of the above, plus very well-written and/or an insightful or important point is made.

–=Criterion is listed or identified, but it is not clearly explained or integrated.

0=Criterion is not identified or addressed, or required information is not provided.

For a grade of A, students must earn a + in all criteria. For a grade of B, students must earn a ✓ in all criteria. An A– or B+ grade is earned based on the number of criteria rated ✓ or +. The grades of B–, C+, or C are earned based on the number of criteria rated ✓, –, or 0. A grade of F is earned if all criteria are rated – or 0. No C– or D grades are given in graduate courses.
