# Supplementary material for: Developing occupational therapy students’ information and historical literacy competencies: an interprofessional collaborative project
Source: J Med Libr Assoc. 2018 Jul 1;106(3):340–51. doi: 10.5195/jmla.2018.332 (PMC6013127; doi:10.5195/jmla.2018.332)
Supplement: Appendix B [file jmla-106-340-s002.pdf]

JMLA Journal of the Medical Library Association

SC

Supplemental content to
J Med Libr Assoc. Jul;106(3):dx.doi.org/10.5195/jmla.2018.332
www.jmla.mlanet.org
© Fleming-Castaldy 2018

Developing occupational therapy students' information and historical literacy competencies: an interprofessional collaborative project

Rita P. Fleming-Castaldy

APPENDIX B

Information and historical literacy learning objectives and instructional methods

Learning objectives	Description of instructional methods
Articulate how the research needed to answer a clinical question differs from the study of history.	Librarian and course instructor review the differences between historical inquiry and clinical research and provide concrete examples in a "show and tell" format.
Become familiar with the course-specific online research guide.	Librarian walks students through the content of the research guide using lab computers; instructor highlights key aspects.
Become familiar with the non-electronic library resources.	Librarian reviews course's extensive print reserves; instructor provides context relevant to students' foci.
Distinguish diverse historical research resources and their distinct content.	Librarian and course instructor highlight the unique characteristics and value of each resource.
Refine database search strategies to effectively access information from historical resources.	Librarian and course instructor give hints on effective search strategies to access historical works (e.g., use the language of a prior time period not today's "key words").
Evaluate whether resources are primary, secondary, or tertiary.	Librarian provides guidelines for distinguishing primary, secondary, and tertiary historical works.
Understand how values impacted the founding of occupational therapy (OT) and its evolution and become familiar with seminal works.	Course instructor facilitates discussion about the relevance of values to OT's development, and students complete a "scavenger hunt" of the literature to find historical quotes about OT values from the 1910s to the 1960s.
Acquire mastery in the use of microfilm to access materials not available electronically or in print.	Librarian teaches students how to use the microfilm reader, and students practice searching journals on microfilm to obtain needed information.
Employ effective search strategies to obtain primary historical information from the course online research guide, print reserves, and microfilm.	Students use electronic, microfilm, and hands-on searches to find primary sources during a lab session, during which the librarian and course instructor are available for guidance. Ongoing support is provided by instructor and library staff.
Compose a coherent literature review, cogent historical analysis, and informed critique of capstone topic.	Assigned readings, active reflections, seminar discussions, and 1:1s promote critical inquiry and informed discourse.<br>Historical literature review and reflective analysis capstone.
