# Supplementary material for: Developing occupational therapy students’ information and historical literacy competencies: an interprofessional collaborative project
Source: J Med Libr Assoc. 2018 Jul 1;106(3):340–51. doi: 10.5195/jmla.2018.332 (PMC6013127; doi:10.5195/jmla.2018.332)
Supplement: Appendix C [file jmla-106-340-s003.pdf]

JMLA Journal of the Medical Library Association

SC

Supplemental content to
J Med Libr Assoc. Jul;106(3):dx.doi.org/10.5195/jmla.2018.332
www.jmla.mlanet.org
© Fleming-Castaldy 2018

Developing occupational therapy students' information and historical literacy competencies: an interprofessional collaborative project

Rita P. Fleming-Castaldy

APPENDIX C

Contents of the leadership in occupational therapy online research guide

Resource	Content examples
Electronic journals: historical journals in occupational therapy and related fields	*Archives of Occupational Therapy:* vol. 1 (1922) to vol. 3 (1924)<br>*Occupational Therapy and Rehabilitation:* vol. 4 (1925) to vol. 30 (1951)<br>*The Modern Hospital:* vol. 1 (1913) to vol. 21 (1923)<br>*Trained Nurse and Hospital Review*: vol. 14 (1895) to vol. 69 (1922)
E-books: rare and historical works that students can access via links provided on the research guide page	*Occupation Therapy: A Manual for Nurses* by Dunton; 1918<br>*Occupational Therapy Applied to Restoration of Function of Disabled Joints* by Baldwin; 1919<br>*Studies in Invalid Occupation: A Manual for Nurses and Attendants* by Tracy; 1914<br>*Teaching the Sick: A Manual of Occupational Therapy and Re-Education* by Barton; 1919<br>*The Work of Our Hands: A Study of Occupation for Invalids by* Hall and Buck; 1915
Links to print and video databases with explanations of scope	Academic Video Online, American Occupational Therapy Foundation Wilma West Library, CINAHL, Cochrane Library, ERIC (EBSCO), Films on Demand, Health and Society in Video, Ingenta, JAMA, JSTOR, MEDLINE, OT Search PubMed, ProQuest Central, ProQuest Health & Medical Complete, PsycINFO, 60 Minutes, Taylor & Francis
List of print reserves and reference books with tips on how to use them for capstone	*Index to the Journal of the American Physical Therapy Association*: use like a book index to search for a subject. Many of the articles will be specific to physical therapy; however, some will be helpful to your research. For example, the subject "cerebral palsy" has a subheading, Equipment. Listed under this subheading there is a citation for an article from 1935, "Toys, Games, and Apparatus for Children with Cerebral Palsy."
List of print and microfilm journals and access directions	The *American Journal of Occupational Therapy:* The library has vol. 1 (1947) to vol. 69 (2015) on microfilm and vol. 6 (1952) to vol. 8 (1954) and vol. 10 (1956) to vol. 71 (2017 up to current issue) in print, shelved with the current periodicals. A librarian can assist you with printing from the microfilm.
Sources, searching, and citation chasing guidance and hints	The differences between historical and research materials is clarified with descriptions and examples of primary, secondary, and tertiary sources. Tips for finding historical sources using the language of prior time periods (e.g., "cripple," "insane") and helpful synonyms are provided. The strategy of using citations in the reference list of a relevant work to track down additional sources, including seminal works, is described.

* Occupational therapy was founded as a profession in 1917. Its founders included a nurse, physician, and social worker; thus, the earliest literature about occupational therapy is found in the literature of these fields.
